# Neurological Complications After Lateral Lumbar Interbody Fusion: Incidence, Patterns, and Risk Factors in a 545-Patient Cohort

**DOI:** 10.3390/neurolint18090178

**Published:** 2026-09-20

**Authors:** Atef F. Hulliel, Dana Saleh, Ahmad Zayd Alkadri, David J. Boughanem, Rafal Chociej, Daven Mejica, Bryan Clampitt, Srujan Kopparapu, Jay Kumar, Patrick Kim, Mohsen Rostami, Puya Alikhani

**Affiliations:** Department of Neurosurgery, Brain and Spine, Morsani College of Medicine, University of South Florida, Tampa, FL 33606, USA; danasaleh@usf.edu (D.S.); azalkadri@usf.edu (A.Z.A.); djb4@usf.edu (D.J.B.); rchociej@usf.edu (R.C.); dmejica@usf.edu (D.M.); baclampitt@usf.edu (B.C.); skopparapu@usf.edu (S.K.); jay.i.kumar.01@gmail.com (J.K.); pkim@usf.edu (P.K.); mohsenrostami@usf.edu (M.R.); palikhan@usf.edu (P.A.)

**Keywords:** lateral lumbar interbody fusion, incidence, morbidity, neurological

## Abstract

**Introduction**: Lateral lumbar interbody fusion (LLIF) provides effective anterior column reconstruction while avoiding extensive posterior dissection; however, its transpsoas trajectory places the lumbar plexus at risk of neurological complications. The incidence, clinical patterns, and predictors of these events remain incompletely defined because of heterogeneous reporting across studies. **Method**: We performed a retrospective cohort study of consecutive adult patients undergoing transpsoas LLIF at a tertiary spine centre between 2016 and 2023. Demographic, clinical, and operative variables were collected, and postoperative neurological complications were identified from clinical documentation and imaging when available. Multivariable logistic regression was performed to determine independent predictors of transient motor weakness, acute thigh paraesthesia, and transient paraesthesia. **Results**: Among 545 patients, 167 (30.6%) experienced at least one neurological complication. The most common events were acute thigh paraesthesia (14.4%) and transient motor weakness (13.2%), whereas hip flexion weakness (2.1%), femoral palsy (1.3%), and psoas haematoma (1.4%) were uncommon. Obesity was independently associated with transient motor weakness (OR 1.77, 95% CI 1.06–2.93; *p* = 0.028). Younger age independently predicted acute thigh paraesthesia (OR 0.96 per year, 95% CI 0.94–0.99; *p* = 0.002) and transient paraesthesia (OR 0.97 per year, 95% CI 0.94–1.00; *p* = 0.049). Multilevel surgery was independently associated with acute thigh paraesthesia (OR 1.67, 95% CI 1.02–2.74; *p* = 0.043). **Conclusions**: Neurological complications following LLIF are relatively frequent but are predominantly transient sensory disturbances or temporary motor weakness. Obesity, younger age, and multilevel surgery identify patients with increased risk of specific neurological events. These findings support individualized counselling, careful surgical planning, and continued investigation into anatomical and intraoperative factors underlying LLIF-related neurological morbidity.

## 1. Introduction

Lateral lumbar interbody fusion (LLIF), described by Ozgur and colleagues in 2006, has become a widely adopted minimally invasive technique for anterior column reconstruction of the lumbar spine [1]. By approaching the disc space through a retroperitoneal, transpsoas corridor, LLIF permits placement of a large-footprint interbody cage that rests on the dense apophyseal ring, indirect decompression of the neural elements, and correction of coronal and sagittal malalignment without the morbidity of a formal anterior exposure or extensive posterior dissection [2,3]. These advantages have contributed to the widespread adoption of LLIF across a broad range of degenerative and deformity-related spinal conditions, particularly in the ageing population, where the increasing prevalence of lumbar degenerative disease and adult spinal deformity represents a significant burden on health-related quality of life [4,5].

Despite these advantages, the transpsoas approach carries a complication profile that reflects the intimate relationship between the surgical corridor and the lumbar plexus. Cadaveric studies have demonstrated that the nerves of the plexus lie within and dorsal to the psoas muscle and migrate anteriorly at the caudal lumbar levels, leaving only a limited safe working zone at L4–5 [6,7]. Traversal and retraction of the psoas may therefore injure motor and sensory branches, manifesting as anterior thigh pain, dysaesthesia, sensory disturbance, and hip flexor or quadriceps weakness [8,9]. A systematic review of 6819 patients reported transient neurological events in approximately 36% of cases, although persistent deficits were considerably less frequent [10]. Reported incidences nevertheless vary widely between series, largely because of heterogeneous definitions, follow-up intervals, and methods of ascertainment [11].

The mechanisms and risk factors underlying these events remain incompletely defined. Intraoperative neuromonitoring, including triggered and free-run electromyography, was introduced to reduce plexus injury, yet postoperative deficits continue to occur despite its use [12]. Patient- and procedure-level factors such as obesity, patient age, the number of levels treated, and the use of supplemental posterior fixation have each been proposed as contributors, but the supporting evidence is inconsistent and frequently derived from small cohorts [3,9]. A clearer understanding of which patients are at greatest risk, and of which specific neurological syndromes are associated with which factors, would inform both preoperative counselling and intraoperative decision-making.

We therefore undertook a large single-centre retrospective cohort study to characterise the incidence and patterns of neurological complications following LLIF, and to identify independent demographic and operative risk factors for the most common of these events. To date, most studies have reported a single composite neurological outcome, which may obscure factors specific to individual syndromes; evidence addressing the risk factors for distinct neurological syndromes therefore remains limited. The aim of this study was to identify risk factors for specific neurological syndromes rather than a composite neurological outcome.

## 2. Materials and Methods

### 2.1. Study Design and Patient Selection

This was a retrospective cohort study conducted at a single tertiary spine centre from 2016 to 2023. The study was designed, conducted, and reported in accordance with the Strengthening the Reporting of Observational Studies in Epidemiology (STROBE) statement for cohort studies. The study protocol was approved by the Institutional Review Board (No. Pro00023643), which waived the requirement for individual informed consent given the retrospective design. Consecutive adult patients (aged ≥ 18 years) who underwent a retroperitoneal transpsoas lateral lumbar interbody fusion for degenerative and deformity-related lumbar pathology between 2016 and 2023 were eligible for inclusion. Patients with incomplete operative or clinical records that precluded reliable ascertainment of the study outcomes were excluded. The final cohort available for analysis comprised 545 patients; the number of patients contributing to each specific outcome varied according to the completeness of the corresponding clinical or imaging documentation. Patients were followed postoperatively with routine clinical and, where indicated, radiographic review; the minimum duration of follow-up was 24 months. Postoperative neurological assessment was performed by the operating surgical team during the index hospital admission and at scheduled outpatient reviews, undertaken at 2 to 6 weeks and at 3, 6, 12, and 24 months after surgery, with additional visits arranged as clinically indicated. The neurological examination together with any sensory or motor symptoms was recorded at each encounter, and an event was classified as transient or persistent according to whether the documented deficit had resolved on subsequent review. To limit selection and ascertainment bias, all consecutive eligible patients treated during the study period were enrolled, and the study outcomes were defined a priori and identified from standardised clinical documentation supplemented by imaging where available. Of the patients who were otherwise eligible, 12 patients were excluded because incomplete operative or clinical records precluded reliable ascertainment of the study outcomes.

### 2.2. Data Extraction

Demographic and clinical variables were extracted from the electronic medical record, including age, sex, and body-mass index (BMI), together with relevant comorbidities and any history of prior lumbar surgery. Operative variables included the number of levels treated and the surgical approach. For analysis, BMI was dichotomised at 30 kg/m^2^ (non-obese, <30 kg/m^2^; obese, ≥30 kg/m^2^); the number of treated levels was categorised as single-level or multi-level (≥2 levels); and age was analysed both as a continuous variable and by group (<65 years and ≥65 years). The approach variable distinguished a combined anterior–posterior procedure from stand-alone lateral surgery.

### 2.3. Outcomes

The primary outcomes were neurological complications occurring in the postoperative period, comprising femoral palsy, hip flexion (iliopsoas) weakness, transient motor weakness, acute thigh paraesthesia, transient paraesthesia, and psoas haematoma. A composite “any neurological complication” outcome was defined as the occurrence of at least one of these events. Neurological complications were diagnosed clinically on the basis of the postoperative examination and, where indicated, confirmed using plain radiographs and computed tomography (CT); psoas haematoma was confirmed on cross-sectional imaging in the subset of patients who underwent CT. For the purposes of this study, acute thigh paraesthesia was defined as new sensory disturbance (numbness, tingling, burning, or dysaesthesia) localised to the anterior or anterolateral thigh and identified on the immediate postoperative examination during the index hospital admission. Transient paraesthesia was defined as postoperatively documented sensory disturbance, in any lower-limb distribution, that subsequently resolved on follow-up assessment. Transient motor weakness was defined as a new, approach-related reduction in hip flexion or knee extension strength on postoperative examination that recovered on subsequent assessment. A neurological event was classified as transient when complete resolution of the symptom or deficit was documented within 6 months of surgery; symptoms or deficits persisting beyond this interval were classified as persistent. Complication severity was additionally considered in relation to the Clavien–Dindo classification of surgical complications. Within this framework, the transient sensory disturbances and self-limiting motor weakness that accounted for the majority of events in the present cohort are broadly consistent with Grade I complications, defined as any deviation from the normal postoperative course that does not require pharmacological treatment or surgical, endoscopic, or radiological intervention, whereas any event necessitating a specific intervention would be assigned to a higher grade.

### 2.4. Statistical Analysis

Continuous variables are reported as mean ± standard deviation and were compared between groups with the Mann–Whitney U test. Categorical variables are reported as number (%) and were compared with Fisher’s exact test. Univariate logistic regression was performed for each candidate predictor. For the three most frequent transient neurological outcomes, transient motor weakness, acute thigh paraesthesia, and transient paraesthesia, multivariable logistic regression models were constructed, incorporating age (per year), sex, obesity, prior surgery, number of levels, and approach type; age and BMI were included a priori as adjustment variables. Associations are expressed as odds ratios (OR) with 95% confidence intervals (CI). Model fit was summarised using the pseudo-R^2^ statistic and the Akaike information criterion (AIC). A two-sided *p* value of less than 0.05 was considered statistically significant. Analyses were performed using IBM SPSS Statistics for Windows, version 26.0. No formal adjustment for multiple comparisons was applied; accordingly, associations of borderline statistical significance should be interpreted with caution.

## 3. Result

### 3.1. Overall Incidence of Neurological Complications

Among the 545 patients analysed, at least one neurological complication was recorded in 167 (30.6%). The most frequent individual events were acute thigh paraesthesia (14.4%) and transient motor weakness (13.2%), followed by transient paraesthesia (7.8%). More serious motor events were uncommon: hip flexion weakness occurred in 2.1% of patients and femoral palsy in 1.3%. Psoas haematoma was identified in 1.4% of the patients who underwent cross-sectional imaging (Table 1). The median duration of postoperative follow-up was 26 months (range, 24–48 months).

### 3.2. Neurological Complications by Number of Levels Treated

Rates of most neurological complications were numerically higher after multi-level than after single-level surgery, including the composite outcome (34.2% vs. 27.9%), but none of these differences reached statistical significance. The strongest trend was observed for hip flexion weakness (3.6% vs. 1.0%; *p* = 0.061) (Table 2).

### 3.3. Neurological Complications by BMI Group

Obesity was associated with a significantly higher rate of transient motor weakness (16.7% vs. 10.3%; *p* = 0.039) and of any neurological complication (37.6% vs. 25.0%; *p* = 0.002). Rates of acute thigh paraesthesia and transient paraesthesia did not differ significantly between obese and non-obese patients (Table 3).

### 3.4. Neurological Complications by Age Group

Younger patients (<65 years) had significantly higher rates of acute thigh paraesthesia (17.6% vs. 10.4%; *p* = 0.043) and transient paraesthesia (10.4% vs. 4.8%; *p* = 0.039) than patients aged ≥ 65 years. In contrast, transient motor weakness and the composite outcome did not differ significantly between the age groups (Table 4).

### 3.5. Independent Risk Factors on Multivariable Analysis

In the multivariable model for transient motor weakness (*n* = 542), obesity was the only independent predictor (OR 1.77, 95% CI 1.06–2.93; *p* = 0.028); age, sex, prior surgery, number of levels, and approach type were not significantly associated (Table 5). For acute thigh paraesthesia (*n* = 537), younger age (OR 0.96 per year, 95% CI 0.94–0.99; *p* = 0.002) and multi-level surgery (OR 1.67, 95% CI 1.02–2.74; *p* = 0.043) were independent predictors (Table 6). For transient paraesthesia (*n* = 538), younger age was the only independent predictor (OR 0.97 per year, 95% CI 0.94–1.00; *p* = 0.049) (Table 7). The models explained a modest proportion of the variance in outcome (pseudo-R^2^ 0.013–0.029).

## 4. Discussion

In this single-centre cohort of 545 patients undergoing transpsoas LLIF, at least one neurological complication was recorded in just under one-third of patients, but the overwhelming majority of these events were transient sensory or motor disturbances rather than structural nerve injuries. Acute thigh paraesthesia and transient motor weakness were the dominant syndromes, whereas femoral palsy, hip flexor weakness, and psoas haematoma were each uncommon. On multivariable analysis, obesity independently predicted transient motor weakness, younger age predicted both thigh and transient paraesthesia, and multi-level surgery predicted acute thigh paraesthesia. These associations were statistically supported but explained only a small fraction of the overall variance, underscoring that neurological events after LLIF are multifactorial.

Our overall neurological complication rate of 30.6% falls within the broad range described in the literature. In one systematic review of 6819 patients, Hijji et al. reported a pooled transient neurological event rate of approximately 36%, with persistent deficits in fewer than 4% [10]. Single-institution series have reported figures ranging from below 10% to more than 60%, a variability that is driven largely by differences in how thigh symptoms are defined and ascertained; using patient-completed pain diagrams, Cummock et al. documented thigh symptoms in 62.7% of patients, most of which resolved within three months [8]. The comparatively lower incidence observed in the present series is likely attributable to the use of clinically documented events rather than prospectively collected symptoms, a methodological difference that has consistently been recognized as an important source of heterogeneity across studies [10]. Our findings reinforce the now well-established view that, although neurological events are common after LLIF [13], the great majority are self-limiting [14].

The predominance of sensory over motor syndromes, and of transient over persistent deficits, accords with the anatomical basis of the transpsoas approach. Cadaveric and imaging studies have consistently shown that the lumbar plexus lies within the substance of the psoas and dorsal to the posterior quartile of the vertebral body, migrating ventrally at more caudal levels and progressively narrowing the safe working corridor at L4–5 [6,7,15,16].

The genitofemoral nerve, which courses on the ventral surface of the psoas and is particularly vulnerable at L3–4 and L4–5, is the presumed substrate for much of the anterior thigh dysaesthesia observed postoperatively, whereas retraction of the muscle belly and its motor branches accounts for the transient iliopsoas and quadriceps weakness [8,17,18]. Serious motor events such as femoral palsy were rare in our cohort is consistent with series in which permanent motor deficits occurred in only a small minority of patients and in which iliopsoas weakness, when it occurred, typically recovered over subsequent months [9,19,20]. Direct measurement of hip flexion strength after LLIF has similarly demonstrated an early, largely approach-related decrement that improves with time.

Obesity emerged as an independent predictor of transient motor weakness, increasing the odds by approximately three-quarters, and was also associated with a higher rate of any neurological complication on bivariate analysis. Several plausible mechanisms may underlie this observation. A greater soft-tissue depth lengthens the transpsoas working corridor and may necessitate deeper and more prolonged retraction, thereby increasing the mechanical and ischaemic burden on the plexus; obese patients also frequently exhibit bulkier psoas, within which the plexus can be more difficult to avoid. The literature on obesity and lateral fusion is, however, mixed. Early experience by Rodgers et al. found no increase in overall early complications among obese patients undergoing extreme lateral interbody fusion, in contrast to the well-documented excess morbidity of open posterior fusion in this group [21]. More recent work has suggested that elevated BMI is associated with an increase in early (90-day) postoperative complications after anterior and lateral approaches, without a corresponding increase in intermediate-term events [22]. Our data, which isolate a specific and predominantly transient motor syndrome, are compatible with the view that obesity principally amplifies approach-related neurapraxia rather than the risk of durable injury.

Perhaps the most unexpected finding was the association between younger age and an increased risk of both thigh symptoms and transient paresthesia. Several mechanisms may potentially explain this observation. Younger individuals often have a larger and more robust psoas muscle with less age-related atrophy, which may reduce the available working corridor and make atraumatic mobilization of the lumbar plexus more challenging. In addition, preserved neural integrity in younger patients may result in greater sensitivity to mechanical manipulation or retraction. Conversely, older patients may exhibit age-associated psoas atrophy, potentially allowing easier access through the transpsoas corridor and reducing neural strain during LLIF [23,24,25]. However, psoas morphology and neural calibre were not assessed in the present cohort; therefore, these potential mechanisms remain speculative and should be considered hypotheses for future imaging-based studies rather than established explanations for the observed association.

Multi-level surgery independently predicted acute thigh paraesthesia, consistent with a dose–response relationship between the extent of psoas manipulation and the likelihood of sensory disturbance. Each additional level treated entails further docking, dilation, and retraction, cumulatively increasing exposure of the plexus to mechanical and ischaemic stress [17,26]. Although rates of most complications were numerically higher after multi-level surgery in our bivariate analyses, only thigh paraesthesia reached significance after adjustment, and the composite outcome did not, suggesting that the incremental risk conferred by additional levels is real but modest. The surgical approach variable was not independently associated with any of the three transient syndromes, indicating that, within this cohort, the addition of posterior fixation did not materially alter the risk of these predominantly approach-related neurological events. To address the possibility that pooling two different procedures might bias the findings, approach type (stand-alone lateral versus combined anterior–posterior) was entered as a covariate in each multivariable model; it was not independently associated with any of the transient neurological syndromes, making it unlikely that combining the two procedures materially confounded the reported associations.

These observations have implications for both intraoperative strategy and neuromonitoring. Triggered and free-run electromyography were introduced to localise the plexus and limit retraction-related injury, and prospective work has shown that discrete-threshold electromyography can identify the posterior location of neural structures during the approach [12,27]. Nevertheless, electromyography-based monitoring has repeatedly proved insufficient to prevent all deficits, and significant postoperative weakness has been reported despite unremarkable intraoperative recordings [18,28]. This limitation has motivated interest in multimodal strategies incorporating motor evoked potentials and saphenous somatosensory evoked potentials to monitor femoral nerve function, which some series suggest may reduce postoperative deficits, although the evidence base remains limited and heterogeneous [29,30]. Our finding that most neurological events are transient does not diminish the value of these measures, but it does temper expectations that monitoring alone will abolish a complication profile that is, in substantial part, an anticipated consequence of traversing the psoas.

Taken together, our results support a pragmatic, patient-specific approach to preoperative counselling and operative planning. Patients, particularly those who are obese, younger, or scheduled for multi-level surgery, should be counselled that transient thigh sensory symptoms and motor weakness are common, but that persistent deficits are infrequent and that most symptoms resolve over the ensuing weeks to months. Where feasible, minimising retraction time, judicious level selection, attention to psoas morphology on preoperative imaging, and the use of multimodal neuromonitoring represent reasonable measures to mitigate risk. The modest explanatory power of our models, however, indicates that much of the variation in neurological outcome remains unaccounted for by the measured factors and is likely to reflect intraoperative variables, retraction time, treated level, cage dimensions, and surgeon experience, that were not captured here and that warrant prospective study.

This study has several limitations. First, its retrospective, single-centre design introduces the potential for selection and ascertainment bias, and reliance on clinically documented events almost certainly underestimates the true incidence of transient sensory symptoms relative to prospective, patient-reported diaries, which may in part explain our lower overall rate compared with some published series. Second, the number of patients contributing to individual outcomes varied, reflecting missing data; psoas haematoma in particular was assessed only among patients who underwent cross-sectional imaging, so its reported incidence may not generalise to the whole cohort. Third, the multivariable models explained only a small proportion of the variance in outcome, indicating that important unmeasured confounders, including cage size, intraoperative neuromonitoring modality, and surgeon experience, were not included. Finally, the analysis of several outcomes across multiple subgroups raises the possibility of type I error, and the findings, particularly the age associations, should be regarded as hypothesis-generating and confirmed in prospective cohorts.

The study also has notable strengths. It comprises a large, consecutive, single-centre cohort of more than 500 patients treated within a consistent institutional protocol, which reduces between-surgeon and between-institution heterogeneity and enhances the internal validity of the risk-factor analyses. Mechanical and haematoma-related events were confirmed with radiographs and CT rather than inferred from clinical suspicion alone. Both bivariate and adjusted analyses were undertaken, allowing clinically relevant demographic and operative factors to be evaluated while controlling for age and BMI. Finally, as a real-world consecutive series, the cohort reflects routine practice and offers findings that are readily generalisable to contemporary LLIF patients.

The generalisability of these findings should nevertheless be considered in the context of the study setting. The cohort was treated at a single high-volume tertiary spine centre, and the observed incidences and associations may reflect the patient population, surgical techniques, retraction and neuromonitoring practices, and perioperative pathways used at this institution. Accordingly, the findings are most directly applicable to contemporary transpsoas LLIF performed in similar clinical settings, and further evaluation in multicentre cohorts would help determine their broader applicability.

## 5. Conclusions

In this large single-centre cohort of patients undergoing transpsoas LLIF, neurological complications occurred in approximately one-third of patients, but most events represented transient sensory symptoms or temporary motor weakness rather than permanent neurological injury. Acute thigh paraesthesia and transient motor weakness were the predominant manifestations, whereas severe complications such as femoral palsy and psoas haematoma were uncommon. Obesity independently increased the risk of transient motor weakness, while younger age and multilevel surgery were associated with higher rates of sensory disturbances. These findings highlight that neurological morbidity after LLIF is multifactorial and cannot be fully predicted by conventional demographic and operative variables alone. Given the retrospective design, the reliance on clinically documented outcomes, and the modest explanatory power of the multivariable models, these risk-factor associations should be regarded as exploratory and hypothesis-generating rather than definitive. Although neuromonitoring and refined surgical techniques remain essential, prevention strategies should incorporate patient-specific anatomical considerations, operative planning, and realistic preoperative counselling regarding expected recovery patterns. Prospective multicentre studies incorporating detailed anatomical measurements, intraoperative parameters, and patient-reported outcomes are warranted to improve risk prediction and further reduce LLIF-associated neurological morbidity.

## Figures and Tables

**Table 1:** Overall Neurological Complication Incidence.

Complication	n/N	Incidence
Femoral palsy	7/526	1.3%
Hip flexion weakness	11/526	2.1%
Transient motor weakness	72/545	13.2%
Acute thigh paresthesia	78/540	14.4%
Transient paresthesia	42/541	7.8%
Psoas hematoma	4/292	1.4%
Any neurological	167/545	30.6%

**Table 2:** Neurological Complications by Number of Levels.

Complication	Single-Level	Multi-Level	*p*-Value
Femoral palsy	2/302 (0.7%)	5/224 (2.2%)	0.142
Hip flexion weakness	3/302 (1.0%)	8/224 (3.6%)	0.061
Transient motor weakness	39/308 (12.7%)	33/237 (13.9%)	0.761
Acute thigh paresthesia	38/306 (12.4%)	40/234 (17.1%)	0.159
Transient paresthesia	23/307 (7.5%)	19/234 (8.1%)	0.914
Any neurological	86/308 (27.9%)	81/237 (34.2%)	0.140

**Table 3:** Neurological Complications by BMI Group.

Complication	Non-Obese	Obese	*p*-Value
Transient motor weakness	31/300 (10.3%)	41/245 (16.7%)	0.039
Acute thigh paresthesia	41/297 (13.8%)	37/243 (15.2%)	0.730
Transient paresthesia	22/298 (7.4%)	20/243 (8.2%)	0.838
Any neurological	75/300 (25.0%)	92/245 (37.6%)	0.002

**Table 4:** Neurological Complications by Age Group.

Complication	Age < 65	Age ≥ 65	*p*-Value
Acute thigh paresthesia	32/182 (17.6%)	26/250 (10.4%)	0.043
Transient paresthesia	19/182 (10.4%)	12/251 (4.8%)	0.039
Transient motor weakness	22/184 (12.0%)	34/253 (13.4%)	0.754
Any neurological	62/184 (33.7%)	67/253 (26.5%)	0.127

**Table 5:** Multivariable Logistic Regression, Transient Motor Weakness.

Variable	OR	95% CI	*p*-Value
Age (per year)	1.00	0.98–1.03	0.983
Male sex	1.21	0.73–2.01	0.465
Obese (BMI > 30)	1.77	1.06–2.93	0.028
Prior surgery	0.96	0.57–1.59	0.863
Multi-level (≥2)	1.11	0.67–1.84	0.692
AP approach	1.11	0.67–1.86	0.685

*Obesity independently predicted transient motor weakness (OR 1.77, p = 0.028). n = 542, Pseudo R^2^ = 0.013, AIC = 433.0.*

**Table 6:** Multivariable Logistic Regression, Acute Thigh Paresthesia.

Variable	OR	95% CI	*p*-Value
Age (per year)	0.96	0.94–0.99	0.002
Male sex	0.89	0.54–1.46	0.641
Obese (BMI > 30)	1.09	0.66–1.78	0.740
Prior surgery	0.98	0.60–1.62	0.946
Multi-level (≥2)	1.67	1.02–2.74	0.043
AP approach	0.80	0.49–1.32	0.392

*Younger age (OR 0.96/year, p = 0.002) and multi-level surgery (OR 1.67, p = 0.043) independently predicted thigh paresthesia. n = 537, Pseudo R^2^ = 0.029, AIC = 446.1.*

**Table 7:** Multivariable Logistic Regression, Transient Paresthesia.

Variable	OR	95% CI	*p*-Value
Age (per year)	0.97	0.94–1.00	0.049
Male sex	0.76	0.40–1.47	0.417
Obese (BMI > 30)	1.11	0.58–2.13	0.746
Prior surgery	1.02	0.53–1.97	0.947
Multi-level (≥2)	1.15	0.60–2.21	0.673
AP approach	1.03	0.53–1.98	0.937

*Younger age was the only independent predictor (OR 0.97/year, p = 0.049). n = 538, Pseudo R^2^ = 0.016, AIC = 299.2.*

## Data Availability

The data presented in this study are available from the corresponding author upon request. The data are not publicly available because they contain protected health information and are subject to institutional privacy and ethical restrictions.

## Abbreviations

The following abbreviations are used in this manuscript:

LLIF	Lateral Lumbar Interbody Fusion
BMI	body-mass index
CT	computed tomography
